# Microfluidic Technology for the Generation of Cell Spheroids and Their Applications

**DOI:** 10.3390/mi8040094

**Published:** 2017-03-23

**Authors:** Raja K. Vadivelu, Harshad Kamble, Muhammad J. A. Shiddiky, Nam-Trung Nguyen

**Affiliations:** 1 School of Natural Sciences, Nathan Campus, Griffith University, 170 Kessels Road, Brisbane, QLD 4111, Australia; raja.vadivelu@griffithuni.edu.au (R.K.V.); m.shiddiky@griffith.edu.au (M.J.A.S.); 2 QLD Micro- and Nanotechnology Centre, Nathan Campus, Griffith University, 170 Kessels Road, Brisbane, QLD 4111, Australia; harshad.kamble@griffithuni.edu.au

**Keywords:** microfluidics, bioMEMS, cell spheroids, three-dimensional cell culture, tissue engineering

## Abstract

A three-dimensional (3D) tissue model has significant advantages over the conventional two-dimensional (2D) model. A 3D model mimics the relevant in-vivo physiological conditions, allowing a cell culture to serve as an effective tool for drug discovery, tissue engineering, and the investigation of disease pathology. The present reviews highlight the recent advances and the development of microfluidics based methods for the generation of cell spheroids. The paper emphasizes on the application of microfluidic technology for tissue engineering including the formation of multicellular spheroids (MCS). Further, the paper discusses the recent technical advances in the integration of microfluidic devices for MCS-based high-throughput drug screening. The review compares the various microfluidic techniques and finally provides a perspective for the future opportunities in this research area.

## 1. Introduction

In the past decade, cell-based assays have undergone a noticeable transition from two-dimensional (2D) to three-dimensional (3D) cell culture. A cell culture is the basic tool for drug discovery, investigation of the mechanism of diseases, and tissue engineering. A 3D cell culture maintains the significant physiological relevance of cell-based assays [1]. A 3D cell culture mimics the sophisticated in-vivo environment which is crucial for efficiently predicting the mechanisms of drug action before clinical trials. Traditionally, 2D cell cultures on a flat substrate are employed as in-vitro models, because they are inexpensive and more accessible than animal models. However, 2D culture models may not be able to mimic the in-vivo systems in terms of cellular physiology, metabolism and protein expression (e.g., membrane proteins). Current literature indicates that the spatially confined 2D cultures attribute to the forced inhabitation of cells grown on a flat and rigid surface [2]. The flat surface requires cytoskeleton to establish contact between neighbouring cells and exert artificial polarity [3]. Thus, 2D cultures cannot provide adequate extracellular matrix (ECM) formation and promote cell–cell and cell–matrix interaction to form a complex communication network within a tissue-specific architecture [4]. ECM is a critical cellular factor for structural support and biochemical cues that regulate cell proliferation, adhesion and migration. Furthermore, cells in a monolayer are exposed to the bulk of media with sufficient oxygen and nutrients, whereas the response of cells in a 3D tissue to nutrient and soluble factors depends on their diffusion and the corresponding concentration distribution [5,6].

The limitations of 2D culture systems motivate the development of 3D culture. In contrast to the flat 2D culture, a 3D culture consists of multi-cellular layers, which are critical for both biochemical and mechanical characteristics of a tissue. Thus, a 3D construct allows for the optimal transport of nutrient, gas, growth factors and cellular waste similar to in-vivo processes. To date, countless efforts have been reported on the production of more biologically relevant 3D tissue models using both scaffold-based and scaffold-free strategies. Microtissues constructed with scaffold rely on supporting materials, which raises issues of biocompatibility and cell–material biorecognition. Biodegradable scaffold substitutes a large amount ECM, resulting in tissue that is composed of less densely packed cells [7]. Furthermore, biodegradable scaffolds exert sensitivity to standard sterilization method when used as an implant in the surgical site. In contrast, scaffold-free approaches initiate interactions between cells and substrate to maximize cell–cell interaction by self-generated ECM. In recent years, scaffold-free methods have been developed to enable the self-assembly of cells into multi-planar cell sheets or spherical cell colonies, often referred to as multicellular spheroids (MCS). These two scaffold-free 3D constructs can potentially generate their own ECM components.

Holtfreter and Moscona demonstrated the first formation of MCS using self-assembled cells suspension without external forced interaction with a biomaterial [8]. With this technology, MCS became an important 3D model for tissue engineering and drug testing. A multicellular model is attractive because of its simplicity and ability to mimic the native tissue with a closely packed heterogeneous cell population. Compared to a 2D cell culture, MCS poses improved growth kinetics, better biochemical signalling and enhanced physiochemical gradient. Typical MCS generation methods are cell culture on non-adherent surfaces, spinner flasks, rotating reactor and microwell arrays. Despite the advantages mentioned above, conventional methods for growing MCSs have limited performance in terms of standardized reproducibility and size uniformity. Spheroids produced from conventional methods are usually transferred to another platform for functional characterization and drug testing. This process is often laborious and affects the quality of the spheroids. A microfluidic device can provide a solution for this bottleneck, allowing for high-throughput generation and handling of spheroids. The high-throughput platform is an extremely attractive approach for the clinical applications such as preclinical and therapeutic drug testing.

Since the 1990s, the cutting-edge technology of microfluidics has been adopted for cell culture and producing reliable 3D tissue models that are highly complex, reproducible and tuneable [9]. The small liquid volume, as a key advantage of microfluidics, has been utilized for generating MCSs and the associated cell-based assays. A microfluidic platform is robust and provides several vital features for maintaining in-vivo physiology, such as: (i) integrated components for supplying nutrient and removing waste [10]; (ii) concentration gradient generators suitable for drug delivery and efficacy investigation [11]; (iii) integration of multiple cell-handling tasks such as cell positioning [12], trapping [13] and mixing [14]; (iv) low-cost assays for bioanalysis and high-throughput drug screening [15]; and (v) automated processing to replace tedious manual and robotic handling [16]. Droplet-based microfluidics is an emerging branch of microfluidics that enables the production of highly uniform droplets. This technology allows mixing and encapsulating of cells in a single droplet, which is protected by an immiscible liquid phase. With a high surface area to volume ratio, the microdroplets serve as a unique microbioreactors for a high-yield formation of spheroids [17]. Biomaterials such as polymers and colloid particles were used as the supporting substrate [18]. In addition, droplets containing MCS can be precisely positioned in an array for applications such as cytotoxic testing [19]. Such a droplet array is feasible to control administration of a drug and suitable for high-throughput image-based drug screening.

The present review highlights the recent advances in the development of microfluidics-based 3D spheroid culture. The review focuses on: (i) the formation of MCS in a microfluidic system; (ii) optimization of a microfluidic system for 3D culture; and (iii) integrated systems for high-throughput drug screening. Furthermore, the review also compares and discusses the advantages and limitations of various microfluidic techniques and proposes the future research opportunities, especially to address the current challenges in the field of health care.

## 2. Multicellular Spheroids

### 2.1. Formation of Multicellular Spheroids

**Figure 1:**
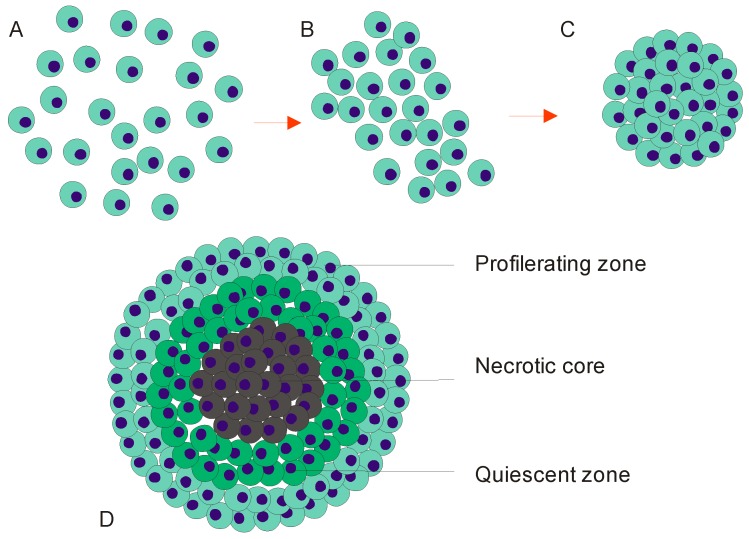
Structure and formation of a multicellular spheroid: (**A**–**C**) formation; and (**D**) structure.

Tissue compaction and cohesion are essential for the spontaneous formation of cell constructs. An engineered spherical tissue should possess viable cells, organized matrix and biomechanical properties to achieve the ultimate biomimicry. Scaffold-free approaches produce tissues by mimicking natural processes that occur during embryogenesis, morphogenesis and organogenesis. If cells are seeded on a planar surface without exogenous material, they inhibit surface adhesion and form clumps in suspension. The two distinct assembly categories are the organization of extracellular matrix and the self-assembly of cells aggregation. The self-assembly process is spontaneous and more pronounced to intrinsic sorting. The process initiates the self-arrangement and cell–cell interactions to organize aggregates in the form of the spheroids or other shapes. Initially, cells aggregate and undergo self-sorting in response to the signal they generate. The cells subsequently form loose clusters. In addition, the self-sorting mechanism allows cells to selectively form a discrete population and segregate from other populations. A number of intercellular adhesion models have been formulated to describe the formation and compaction of MCS. These models mainly consider the three-step process shown in Figure 1: (i) interaction between ECM and integrin to promote cell attachment (Figure 1A); (ii) up-regulation of cadherin upon cell aggregation (Figure 1B); and (iii) homophilic interaction of type-1 transmembrane proteins (E-cadherins) to initiate strong cell adhesion (Figure 1C).

The interaction between ECM and integrin plays an important role as physical linkers to mediate the cells merging process. A long-chain polymerized fibronectin matrix is the major ECM component that links the cells [20]. Mono-dispersed cells make cell–cell contact and enhance ECM generation with multiple tripeptide arginyl-glycyl-aspartic (RGD) motifs. The RGD motifs then bind with integrins to form integrin/ECM fiber-mediated composition on the cell membrane surface [21]. The integrin–ECM interaction is the base for cell binding that eventually promotes a stronger cell–cell adhesion which is essential for the acceleration of cell aggregation (Figure 1A). Subsequently, clusters of the cell aggregates fuse and assemble into a loosely assembled spherical cell structure. This loose aggregate is highly permeable to nutrient and soluble factors. The next process is known as delayed process in which cells establish the cohesion activity. The integration of the integrin–ECM facilitates this process to increase the cell aggregation. Cells in the aggregates are healthy and possess higher survivability [22]. The regulation of cell–cell recognition and interaction at the delayed phase is initiated by E-cadherin (Figure 1B) [23]. The classical role of cadherins is to increase the adhesiveness of the cell–cell contact and provide a structural support.

E-cadherin expression is established to initiate the cell compaction and it is auto regulated until a specific threshold [24]. Furthermore, cadherin molecules bind with each other by homophilic interaction to generate a strong cell cohesion which is connected by adherent junctions [25,26]. Finally, the loose aggregates compact and condense into a spherical tissue, forming a multicellular spheroid. E-cadherin mediated adhesion is an essential factor for the plasticity of cells which allows the cells shape to change upon contraction (Figure 1C). In brief, co-localisation between the E-cadherin and cytoskeleton facilitates the actin filament rearrangement to form a bundles and networks [27].

### 2.2. Properties of Multicellular Spheroid

The cells within a MCS are heterogeneously exposed to the nutrient, growth factors and oxygen supply due to MCS structure. Thus, it is meaningful to engineer MCSs with a pre-defined size to be able to predict and regulate the self-sufficiency of MCSs for survival and homeostasis. The metabolic activities within the MCSs are heavily depended on their ability to maintain a sufficient degree of mass transport. [28]. A relatively thick MCSs with diameters ranging between 150 and 200 µm is typically composed of diffusion-restricted tissue. Oxygen diffusivity in a MCS is one of the critical factor which is commonly measured using O_2_-sensitive microelectrodes [29]. It is important to note that the zone next to the core of a MCS is most likely to have insufficient oxygen supply and therefore it is more susceptible to the decrease in metabolic rate. Moreover, diffusion of nutrient is another hurdle. Alvarez-Perez et al. reported that proton magnetic resonance with pH-sensitive indicator shows a poor diffusion of nutrients within the MCSs [30]. Furthermore, the cell barrier impedes the elimination of waste, and therefore dumps them at the core of the spheroid. The cells at this region lose their biological activity leads to the occurrence cell death and formation of necrotic core [31]. Figure 1D shows the three basic layers of the MCSs: (i) the outer layer containing proliferating cells with active metabolic rate; (ii) the middle layer consisting of cells at quiescent state in which cells may potentially attain higher metabolic rate and proliferate upon exposure to nutrient; and (iii) the inner region containing cells underwent necrosis. Cells at this region suffer from insufficient nutrient and lose their biological activity to excrete waste.

Considering their structure, MCSs seem to be replicating the architecture of a native tumour tissue. In nature, tumour microenvironment in vivo is biologically heterogeneous, comprising of progressive growth and induce metastasis by extensive neovascularization called “tumour angiogenesis” [32]. The angiogenesis in the tumour predominates the cells survival and proliferation by promoting the biochemical mass transport. Unexpectedly, an increase in angiogenesis decreases the drugs response to the therapy. Realizing this, it will be beneficial to generate co-cultured MCS tumour model by using cancer cells and endothelial cells which will ensures heterotypic cell–cell interaction to form a MSC with tumour vascularity [33]. The vascularized tumour spheroid closely resembles the cellular heterogeneity of solid in vivo tumours.

## 3. Conventional Methods for Spheroid Generation

Recently, a wide range of basic and complex methods has been developed to generate MCSs. The most important prerequisite is the ability to control the size and uniformity of MSC formation to maintaining optimal biological functions of the MSCs. The grand challenge for MSC generation is maximizing the cell density in a small volume and generating a spheroids with the size smaller than 150 µm [34]. The small size facilitates the homogenous delivery of oxygen and nutrients from the exterior to the core. Furthermore, the culture method should enable the efficient drugs delivery and ideally mimic the in-vivo efficacy [35]. On the other hand, it is crucial to establish methods with standardization, automation, quality control and validation. Such initiatives will potentially accelerate the large-scale production of well-defined MSC for drug screening purposes. In summary, adaptation to high throughput formats will be cost effective. Some of the conventional methods for the production of MSC are described as follow.

### 3.1. Pellet Culture

**Figure 2:**
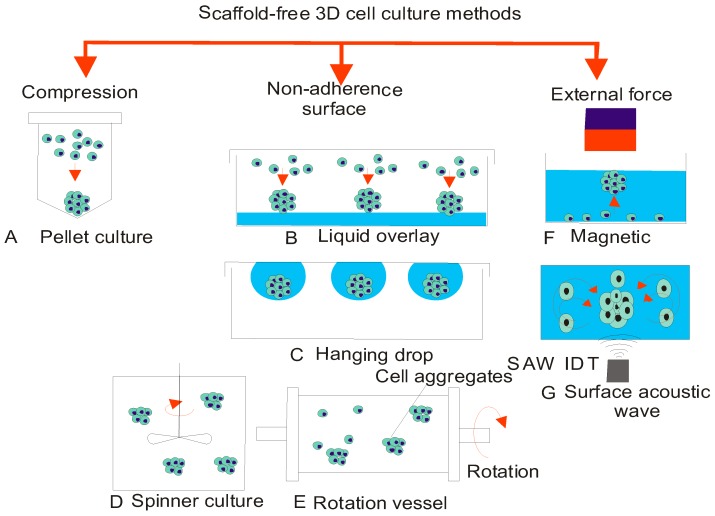
Conventional methods for spheroid generation: (**A**) pellet culture; (**B**) liquid overlay; (**C**) hanging drop; (**D**) spinner culture; (**E**) rotating vessel; (**F**) magnetic force; and (**G**) surface acoustic wave.

Pellet culture is a simple and rapid approach, which was first introduced by Kato et al. [36]. The method employs centrifugal forces to maximize the cell–cell contact and subsequent adhesion at the bottom of a test tube (Figure 2A) with the typical centrifugation acceleration and time as 500 g and 5 min respectively. However, the major drawback of the method is that the shear stress from centrifugation may damage cells and thus provide unreliable results. Another drawback is that this method creates relatively larger spheroids with the diameter of more than 500 µm. Larger MSCs with the diameter range of 500 μm are not suitable for general bioassay development, because oxygen demand in such a large MSC causes hypoxia in the core and thus may not reflect the treatment effectiveness [37]. However, this model is suitable for studying bone regeneration [38]. Typically, low oxygen environment stimulates the differentiation of chondrocytes or chondrogenesis of mesenchymal stem cells [39,40]. Furthermore, this method is not scaled for mass production for high throughput screening and image analysis.

### 3.2. Liquid Overlay

The liquid overlay method allows the cell–cell aggregation instead of the cell adherence by coating the cell culture plate with a non-adherence layer (Figure 2B). This technique was established by Ivascu and Kubbies [41]. A low-adhesive surface was created by coating poly (2-hydroxethyl methacrylate) (pHEMA) on commercially available plates designed with V-shaped or U-shaped bottoms [42]. After seeding cells, a low magnitude of mechanical vibration was applied to promote cell aggregation leading to the MSCs formation. This method is straightforward and easy but suffers from the issues such as reproducibility with sufficiently high yield [43] and non-uniform shape of the spheroids [44]. The usage of different types of culture plates is the main cause for the insufficient reproducibility in size and shape. However, the utility of commercially available Cellstar® Cell-Repellent Surface well plate had shown a high performance [45].

### 3.3. Hanging Drop

The conventional hanging drop culture was first described by Keller for initiating the development of embryonic bodies [46]. This method is based on the sedimentation of the cells due to gravitational force which promotes the cell–cell interaction. These interactions dominate over cell-substrate interaction, leading to the formation of spheroids (Figure 2C). A small droplet containing cell suspension with volume ranging from 20 to 30 µL is seeded onto the lid of polystyrene microwell plate. After turning the plate upside down, the droplets hang and gravity allows the cells to settle at the bottom for self-assembly. This method simplifies the laborious liquid handling processes and has the potential for high throughput. For instance, Tung et al. proposed a 384-well array to generate hanging drops for high-throughput screening [47]. The droplet maintains the shape and rigidly attached to the lid due to the surface tension. The liquid–air interface of the droplet allows gas exchange but is subjected to extensive evaporation [48]. To prevent evaporation, a modified liquid bath reservoir was used to maintain the humidity.

The hanging drop method is applicable for a volume of less than 50 µL because a large volume reduces the role of surface tension against gravity, and the droplet may fall down [49]. Furthermore, owing to the small volumes and the insufficient nutrient supply, a hanging drop cannot sustain a long-term culture. Changing medium by multiple pipetting steps is susceptible to mechanical perturbation which leads to the spreading or collapse of the droplet. Thus, it is not an easy task to add drug or soluble factors to the droplets. However, Frey et al. developed a hanging drop method incorporating a continuous liquid flow network which enables nutrient supply [50]. Hsiao et al. modified the microwell plate with a lid containing ring structures to improve the structural integrity of the hanging drop [51]. The plate is designed with an extra ring for holding more liquid and is more robust against evaporation. Nonetheless, overall conventional hanging drop techniques do not allow real-time imaging to track the formation process of the spheroids. To overcome this limitation, the primary plate of hanging drops are transferred to a secondary plate using transfer and imaging (TRIM) plates [52] or commercially available systems such as InSphero GravityTRAP system. 

### 3.4. Spinning Flasks

Spinning flasks culture is a cell agitation approach based on stirred suspensions. An impeller mixer in the reactor tank prevents cells sedimentation and also promotes cell–cell interaction in the culture medium, Figure 2D. The spinning mechanism allows for sufficient supply of nutrients and soluble factors to cells while facilitating the excretion of wastes [53]. This method is suitable for long term culture. However, the major limitation is the production of non-uniform spheroids. Furthermore, method needs the specialized equipment and consumes a large amount of medium (100–300 mL). Finally, for drug screening purposes, this method requires manual selection of spheroids to obtain a population with similar size [54].

### 3.5. Rotating Vessels

Rotating cell culture bioreactors or rotating wall vessel (RWV) was developed by NASA in 1992 to study cell growth under simulated microgravity condition [55]. Instead of stirring, rotary bioreactor rotates itself to maintain cells in a continuous suspension (Figure 2E). The culture chamber rotates along the horizontal axis to prevent cells to adherence to the chamber wall. The speed of the rotation can be adjusted to exert low shear force, and at the same time to promote the optimal cell–cell adherence for a larger 3D structure. Once 3D aggregates are formed, continuous rotation at a desired speed prevents coalescence of the spheroids. This method enables the development of spheroids with approximately uniform sizes. Rotating vessel method ideally suits for long-term culture of 3D spheroids, because easy replacement of medium allows for efficient supply of nutrient and removal of waste [56]. The main disadvantage of this method is the requirement of specialized equipment.

### 3.6. External Forces

The application of external forces has long been employed to induce cell aggregation and compaction to form 3D structure. Widely used actuation concepts are dielectrophoresis [57], magnetism [58] and acoustic waves [59]. However, these methods are fairly complex, offer little access for visualization of spheroid formation and require specialized equipment. Furthermore, cells are forced to aggregate into a non-uniform geometry, which leads to a heterogeneous morphology and lower yield. These methods can induce mechanical stress onto the cells and may lead to cell damage. Therefore, the formation of spheroids with external forces is not well suited for drug screening. Nevertheless, this approach can meet the demand for 3D culture particularly for cells with lower adherence properties. Recently, magnetic levitation and surface-acoustic-wave have gained increasing attention for the formation and manipulation of spheroids.

#### 3.6.1. Magnetic Levitation

Magnetic levitation was successfully used to construct 3D in-vitro models. This method was used to culture various cell types such as adipocytes, vascular smooth muscle cells and breast tumour cells. Engineering the cell composition and density allows for the formation of the heterogeneous spheroids (Figure 2F). Magnetic levitation was reported to enable tumour and fibroblast cells to interact and form larger spheroids in a shorter time [60]. Moreover, larger spheroids with necrotic cores and region of hypoxia are similar to the in-vivo tumour niche which makes them well suited for the cancer studies. In the study reported by Haisler et al., magnetic nanoparticles were inserted into the layer of 2D confluent cells and then levitated them with an external magnetic field. [61] The magnetic force promotes cell aggregates at the air-liquid interface. The aggregated cell clusters naturally trigger cell–cell interactions. The magnetic nanoparticles are biocompatible and do not induce inflammation or affect the cellular physiology [62]. With this method, cells exhibit improved growth condition, followed by the formation of ECM and compaction. The cohesive multicellular assembly lasted 72 h before forming a spherical shape with a maximum diameter of 1 mm [63]. However, the potential effect of magnetic particles on cellular physiology and metabolism is still not clear and well understood.

#### 3.6.2. Acoustic Wave

Ultrasonic manipulation can be used to concentrate and trap cells in suspension. Bazou et al. observed the changes in the cytoskeleton and adhesion of molecules after cells were exposed to an ultrasound standing wave trap (USWT) (Figure 2G) [64]. Further, a similar approach was employed by Liu et al. to obtain the formation of 3D aggregates of hepatocarcinoma cells. This was followed by the rapid changes in intracellular F-actin within just 30 min [65]. This method is a contactless and can induce larger cell aggregates. The acoustic method provides excellent biocompatibility and contamination-free conditions. This method can be further developed into a tuneable tool to generate MCS. By altering the frequency of the standing wave, spheroids are dynamically assembled and fused into a larger organoids or a desired tissue without using a mould or a template [66]. Controlling the standing surface acoustic wave enables the formation of an acoustic tweezer to precisely pick and assemble cells into an organized 3D structure [67]. More recently, 3D acoustic tweezers were utilized to control and adjust the geometry of spheroid production in only 30 min, achieving a high throughput [68].

## 4. Microfluidic Methods

**Figure 3:**
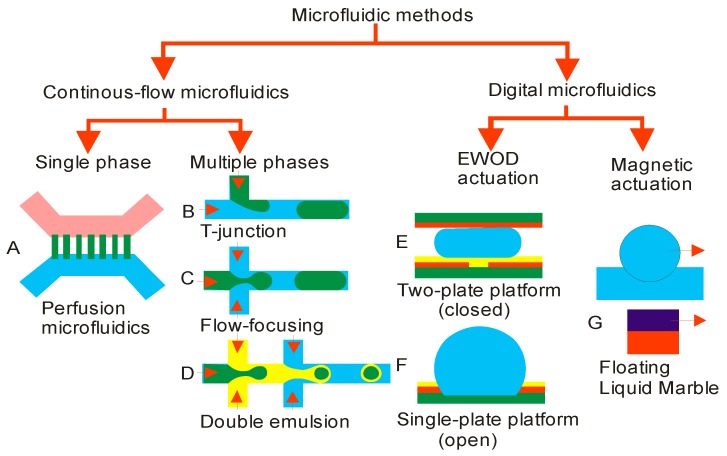
Microfluidics based methods for the generation of spheroids. Single-phase microfluidics: (**A**) perfusion microfluidics; Multi-phase microfluidics; (**B**) T-junction; (**C**) flow-focusing; (**D**) double emulsion—electrowetting on dielectric actuation; (**E**) two-plate platform; (**F**) single-plate platform—magnetic actuation; and (**G**) floating liquid marble.

Microfluidic technology has rapidly evolved in biomedical research as a powerful tool for various applications such as cell-based assay, tissue engineering, molecular diagnostics and drug screening. The basic tasks of microfluidic technology are processing and manipulating small amounts of liquid (10^−9^ to 10^−18^ litters); in a size scale that matches the size of cells and microtissue. Microfluidics is categorized as continuous-flow and digital microfluidics (Figure 3). Continuous-flow microfluidics can be further categorized as single-phase and multi-phase microfluidics. Multi-phase microfluidics and digital microfluidics can accurately and efficiently produce micro droplets within milliseconds enabling a high throughput [69] for cell-based analysis [70]. The implementation of 3D cultures in microscale potentially allows further reduction of the volume of nutrient, reagents, soluble factors and drugs. 3D cell culture with microfluidics provides a higher controllability and provides a cost-effective mean for biomanufacturing. Furthermore, droplet-based microfluidics offers the advantages for cell compartmentalization with a high surface-to-volume ratio. This physical characteristic is desirable in a wide range of molecular and cellular analysis [71]. As the viability of cells during the generation of MCS is critical, a microfluidic platform typically applies a low shear stress and thus minimizes cell damage [72]. Furthermore, an integrated microfluidic device provides a high degree of programmability and configurability. For instance, advance cell-based assays are scalable as an array. This feature allows microfluidics not only to engineer microscale 3D tissues but also more complex tissue system such as artificial organs on a chip. In the following sections, we discuss the state of the art of microfluidic technology for MCS generation and analysis including: (i) platform configuration and applicability; (ii) integrated microfluidics; and (iii) technical limitations and improvement.

### 4.1. Continous-Flow Microfluidics

A continuous flow in microchannels is either delivered by a flow-rate-driven or pressure-driven pumping system. Fluid flow in this small scale is inlaminar regime as surface effects such as friction dominate over volume effects such as inertia. Continuous-flow microfluidics either handles a single-phase or multi-phase segmented flow. Multi-phase flow microfluidics is also often called a droplet-based microfluidics, which generates and manipulates monodispersed microdroplets.

In single-phase continuous-flow microfluidics, the microchannels are coated or filled with hydrogel for trapping and providing a scaffold for cell growth. The technology provides a precise concentration gradient of soluble molecules, nutrients and drugs [73]. Thus, an artificial microenvironment can be created to facilitate spheroid growth (Figure 3A) [74]. In multi-phase continuous-flow microfluidics, liquid droplets are formed and manipulated in a continuous manner. The two established methods for droplet formation are flow-focusing and T-junction configurations (Figure 3B–D). The flow-focusing configuration forms microdroplets by squeezing the liquid stream with two immiscible sheath streams to generate highly monodisperse droplets [75]. The T-junction configuration uses a single sheath flow to break up the dispersed phase into droplets [76]. In both configurations, scaffold materials such as hydrogel can be added into the droplets to support cell growth. 

#### 4.1.1. Single-Phase Microfluidics

Single-phase microfluidic synergies the 3D cell growth and allow the exchange of media by perfusion system. In principle, fluid is continuously flown within a microchannels similar to in-vivo vascularization effect [77]. Conventional systems for spheroid formation and growth is confined to a reactor and isolated from the surrounding. Consequently, spheroid cultivation is limited to short-term culture and is detrimental to the cell viability. This drawback also exists in droplet-based microfluidics, which insufficiently regulates the environment for spheroid growth. To circumvent these limitations, Agastin et al. attempt to grow multiple tumour spheroids using polydimethylsiloxane (PDMS) microbubbles. A physiological flow was established inside the microbubble by media perfusion [78]. This model mimics an in vivo avascular tumour condition. 

Ideally, perfusion method is regarded as a promising platform for anti-cancer drug testing simply because it enhances drug exposure as well as the exchange of nutrients and wastes. In this microfluidic system, media is flown continuously through microchannels and perfuse trapped cells or spheroids. [74]. Thus, the formation and growth of spheroid can be carried out over a long time period (e.g., two weeks) without significant decrease in the cell viability [79]. Moreover, the method allows the possibility of device fabrication with integrated concentration gradient generator for high-throughput applications. [80]. Perfusion flow can be generated simply by gravity and surface tension. In order to increase the throughput, the assay can be scaled up to the 96 wells format. The initial attempt was carried out by Chen et al. developing an integrated micro-capillary network that is connected with supply chambers of culture medium [81]. Following these advances, Sakai et al. designed an improved microwell array with perfused flows to carry out spheroid-based high-throughput drug testing [82]. Another interesting approach was to combine multi-phase and single-phase microfluidics. Spheroids are initially generated in an emulsified droplet, then by displacement of surfactants droplets lead to coalescence and spheroids are exposed to perfusion [83]. Data acquisition and analysis from chemotherapeutic studies showed perfusion-based format showed higher chemoresistivity as compared to the static fluidic environment [84]. Interestingly, this system also allows miniaturizing the combination of both physiologic and pathologic networks such as angiogenesis and thrombosis in a 3D capillary network [85]. For instance, the platform could serve as a model for pathophysiological conditions such as tumour angiogenesis and tissue ischemic model. 

#### 4.1.2. Multi-Phase Microfluidics

In the past five years, several reviews presented a comprehensive overview of methods to form, sort, and merge and manipulate microdroplets for experiments in chemistry and biology [86,87,88]. Recently, droplet-based microfluidics becomes an attractive approach as a microbioreactor to grow and characterize living cells [89] and protocells [90]. Water-in-oil droplets serve as vessels for cell culture. A continuous aqueous flow breaks into droplets and encapsulated by an immiscible phase such as mineral oil with biocompatible surfactants [91]. The immiscible oil phase (W/O), droplets are not optimal for the generation of MCS because they impedes the supply of nutrient and gas exchange [92]. Thus, droplet-based systems only allow for a short-term cell culture. To circumvent these problems, a water-in-oil-in-water (W/O/W) double emulsions (DE) format was used to encapsulate cells. These droplets function as selective barrier to regulate the transport of soluble factors and nutrient. Moreover, the outer aqueous phase ensures the adequate permeability of the oxygen. However, conventional DE generates highly polydisperse droplets which are easily breakable. [93]. The precise control of the surface wettability in the device is critical for the stability of the droplets [94]. The droplet also improves heat and mass transfer and increases the reaction rate. These advantages accelerate the diagnostic results as it allows the molecular or enzymatic reaction to occur in a shorter period of time. Further in the next section, the use of single-phase and multi-phase microfluidics for 3D cell culture is discussed in detail. 

The two main challenges of droplet-based microfluidics are: (i) the formation of biocompatible, monodisperse (<1%–3% dispersity) and stable droplets; and (ii) the real-time observation of cell activity. The size and stability of droplet is critical to sustain long term culture of MCSs as well as drug testing. The hydrodynamic properties such as type of flow, laminar or turbulent, are crucial for scaling of the size of the droplets during emulsification process [95]. The emulsion quality also depends on the viscous shear stress and interfacial tension. The breakup of droplets occurs when viscous shear stress dominates and overcomes the interfacial tension. Further, when generated in bulk, shear force and inertia cause the droplet to coalesce or break [96]. To overcome these limitations, a biocompatible amphiphilic molecule called “surfactant” is absorbed at the interface. Surfactants reduce the interfacial tension between the dispersed and continuous phases, which is critical to maintain the droplet stability and prevent coalescence [97].

The outer oil layer of a droplet serves as a selectively permeable barrier and allows the transport of small molecule across this barrier, which is essential to provide a discrete microenvironment for cell culture. To further enhance cell growth the droplets can be formulated by encapsulating cells with biological additives. Tumarkin et al. demonstrated the possibility of using microgel-based biomaterials, which potentially promoted the cell functionality in terms of enhanced proliferation and adhesiveness for the formation of MCS [98]. For instance, a continuous-flow microfluidic system can be configured to co-encapsulate cells into hydrogel. The gelation process forms the cell-laden microcapsules. Hydrogels are crosslinked with chemical residues by chemical stimuli and also with physical process such as radical reactions, temperature and photon energy [99]. Hydrogel gel particles provide structural support for spheroid growth and function [100,101]. The hydrogel capsules can be scaled up as spheroid carriers to serve as immunoisolation barrier for the cell transplantation [102]. A variety of chemically modified hydrogels have been used to make microcapsules such as alginate-PLL (poly-Llysine)-alginate (APA), thermally responsive hydrogels (agarose, NIPAM based hydrogel and gelatin) and photosensitive hydrogels such as polyethylene glycol (PEG). The precise mechanism of the various types of the encapsulation including cells types, functional outcomes and limitations are summarized is Table 1. 

Biomimetic or biodegradable material based microcarriers are useful for cell culture and drug delivery. Shi et al. modified the method of double emulsion solvent evaporation to fabricate a biodegradable Poly(d,l-lactide) porous microspheres [116]. These microspheres act as a microcarrier to deliver cells which could be used for cell-based therapies. In another study, a double emulsion template was used to encapsulate droplet carrying multiple biomimetic scaffold. The scaffolds consist of the porous cores which acted as a microcarrier and provided a confine environment for growing spheroids. [117]. More recently, a reusable device was developed to customize monodisperse droplets and perform multiple droplet encapsulations [118]. Collectively, it can be concluded that, multiple encapsulation technique holds wider application in a 3D cell based assay.

### 4.2. Digital Microfluidics

A simple platform with easy liquid-handling is critical for lab-on-chip technology. Continuous-flow microfluidics platforms require pumps and tubing for fluid delivery. Thus, handling discrete droplets has advantages over the continuous flow microfluidics. Furthermore, actuation techniques such as magnetic or electric forces are widely used for handling droplets. The digital microfluidics (DMF) is a branch of microfluidics, which integrates the microfluidic devices and electrical forces to manipulate discrete droplets [119,120]. There are two common configurations of DMF for culturing: (i) closed format with droplets sandwiched between two plates (Figure 3E); and (ii) open format with droplets positioned on top of a planar surface (Figure 3F) [121]. The bottom plate usually has an array of actuation electrodes. The top plate made of transparent conductive material allows the optical imaging. For droplet movement with low friction, the surfaces of both plates are coated with hydrophobic material. Droplet manipulation tasks such as dispensing, splitting, merging and coalescence are carried out with electrostatic force by tuning the electric potential of the electrodes [122]. The mobility of the droplet is controlled through a combination of electro wetting (wetting behaviour of liquid) and liquid dielectrophoretic forces (effect of non-uniform electric field on liquid). Thus, DMF offers several advantages over conventional microfluidics such as low cost, portability and low reagent usage, yet provides faster test result. The most significant advantage is the ability to perform multiple biochemical assays simultaneously using a planar array of electrodes [123]. This technology enables the evaluation of several test results in real time.

However, common fluid operation using DMF is restricted to a 2D platform, and therefore it has limitations such as cross contamination, solute adsorption and degradation of soluble factors [124]. Interestingly, the transition from 2D to 3D DMF was demonstrated by submerging droplet in oil between two electrodes. The droplet can be manipulated by moving horizontally and vertically, thus serving as a programmable hanging droplet inside the oil. This platform was successfully used to grow mouse fibroblast [125]. Apart from that, a 3D scaffold based DMF platform was employed by Fiddes et al. to culture NIH-3T3 cells in hydrogel discs [126]. Subsequently, Au et al. improved the scaffold based-DMF to grow HepG2 and NIH-3T3 co-cultured spheroids (organoids) using collagen hydrogels. This method was applied for to hepatotoxicity screening [127]. Recently Aijian et al. demonstrated the adoption of the digital microfluidic in a hanging drop based platform for the generation of spheroids [128]. This method permits automation of liquid handling by dispensing liquid through connected wells to form hanging droplets. This platform is automatable and flexible for liquid handling which enables the formation of hanging drop. These sequential and reconfigurable operations increase throughput for spheroid based assays. 

Our recent approach for multiple spheroids of olfactory ensheathing cells was carried out using floating liquid marble (LM) [129]. A drop of liquid was coated with hydrophobic powder to form an elastic hydrophobic shell with fine pores allowing the exchange of gas. LM also has a low evaporation rate and regulates humidity in floating condition. Moreover, the floating mechanism eases the cell interaction inside the LM due to the internal fluid flow. Additionally, the liquid marble suits as a microbioreactor to generate and to differentiate embroid bodies [130]. Given its advantage of miniaturization, the LM platform allows for the adoption for high-throughput drug screening [131]. Recently, Ooi et al. reported that LM containing ethanol exert self-propelling caused by Marangoni solutocapillary effect [132]. It is also possible to control the locomotion of floating LM by using magnetic actuation (Figure 3G). In the study by Khaw et al., magnetic particles were added to the LM and a moving permanent magnet was used to drag the LM [133]. The actuation of LM is crucial and brilliant for engineering controllable and tuneable functions. LM composed with magnetic nanoparticles can generate centrifugal force to function as microcentrifuge [134]. Additionally, another study addresses the usage of magnetism to split LM with lycopodium−iron oxide [135]. In summary, it is evident that LM can be utilized to support a digital microfluidic platform for 3D cell-based application.

The combination of microfluidics and optics results in a unique technology called optofluidics. The initial optical applications in microfluidic domain were optical tweezers [136] and optical vortex [137]. Photoconductivity can be applied to conventional (DMF) to actuate droplets [138,139] using opto-electro-wetting (OEW) [140]. The technology has shown potential for achieving higher control accuracy by combining photosensitive surfactants and the laser. More recently, 3D droplet manipulations was demonstrated by fabricating a single-sided continuous opto-electro-wetting (SCOEW) platform, which is supposed to have advantage over conventional EWOD and OEW [141]. Further, digitalizing and integrating of the microfluidics with optofluidics offers a powerful imaging solution for biomedical imaging. For instance, the digital holographic microscope is lens free and facilitates real-time imaging three-dimensional tomography imaging of transparent PDMS opto-microfluidic channel [142]. Efforts to utilize this technology with microfluidic include 3D sensing of microorganism [143] and automated cell viability detector [144]. However, application for 3D cell culture has not been yet explored. 

## 5. Application of Spheroids in Microfluidic

### 5.1. Organ Printing

**Figure 4:**
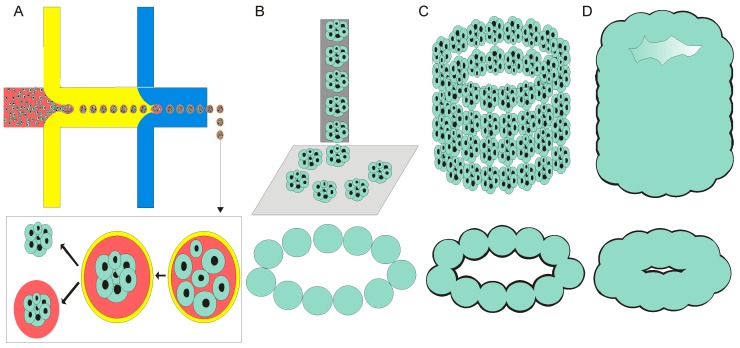
Schematic presentations of principles of 3D tissue spheroids printing: (**A**) microfludic based fabrication of spheroid; (**B**) nozzle is used to dispense spheroids; (**C**) continuous dispensing form layer-by-layer tissue spheroid; and (**D**) layer-by-layer tissue spheroid fusion and bio-assembly of tubular tissue construct.

Organ printing is promising to transform tissue engineering into customized organ biofabrication. Spheroids make an excellent candidate for organ bioassembly. A spheroid has an ideal geometry and may serve as “bioink” for bioprinting. Printing the spheroids layer-by-layer for tissue constructions is a common approach in biopriniting. With the advance computer-aided robotic bioprinting technology, the predefined structure can be precisely printed to achieve desired organ/tissue assembly. The positioning and the placement of the dispersed spheroids are critical factors to achieve a controllable fusion in a 3D tissue. Most recently, Moldovan et al. reported a latest invention called the “Kenzan” method, which utilises microneedles for the spheroids assembly [145]. This technique holds the precision up to micron-level and is able to link the spheroid closely. Further, the array of tightly aligned spheroid undergoes a fusion process to form a complex tissue and syntheses their own ECMs. Tissue construction using spheroid requires a large quantity of uniformly sized spheroids which is required to achieve bio printing with satisfactory resolution. Importantly, a scalable spheroid fabrication method is crucial for producing large quantity of homogenous spheroids. Ultimately, a precise 3D tissue print is achievable by the use of microfluidic based spheroid bio fabricator. Further, application of droplet based digital microfluidic may offer a scalable production of the spheroids at high yield [146]. Figure 4 describes the integration of microfluidic technology for spheroid based 3D printing.

Many other technologies can be considered to improve the use of spheroids for bioprinting as well as tissue fabrication. For instance, a bioprintable scaffold such as electrospun matrix can accurately pattern printed spheroids into desired tissue construct [147]. Furthermore, magnetic 3D printing has the potential to achieve a precise and rapid construction of 3D tissue. For example, a recent study reported a spheroid patterning technique using superparamagnetic iron oxide nanoparticles (SPIONs) for bioprinting [148]. Another approach is using magnetic levitation to construct tumour spheroids which closely mimic native microenvironment [149]. The mechanism of spheroid fusion involves 3D cell–cell interaction and critical for the formation of larger tissue. To date, only a few studies revealed the mechanism of spheroid fusion. Quantification of fusion kinetics, accounting the time lapse for the coalesce of two spheroids is necessary [150]. Recently, Munaz et al. demonstrated a microfluidic platform which can be used to study spheroid fusion as well as drug screening to promote fusion [151]. However, more sophisticated platforms are needed to quantify the cellular parameters such as mechanical strength of fused spheroids.

### 5.2. Organ-On-Chip

To date, the microfluidics-based lab-on-a-chip technologies are facing challenges to reduce the costs and increase the efficiency for drug screening and development. However, with further development it may potentially serve as a tool for preclinical models for human efficacy and safety. Prominently, this technology contributes towards an alternative step to reduce the extensive use of animal testing. Further, the technology allows for mimicking the complexity of animal-based testing models. The combination of fluid physics with 3D cell compartmentalization has gained popularity as organ-on-chip devices. Interestingly, the organ-on-chip concept simplifies clinical bioanalysis by integrating realistic organ models in a single device [152,153].

Initially, the organ-on-a-chip concept was established by combining the cultures of the liver spheroid and neurospheres in a separate chamber and connecting them by a microfluidic circuit [154]. Subsequently, a scaled-up organ-on-chip device was fabricated by combining spheroids grown in arrays and perfused with culture media, which resulted in spheroids fusion and tissue formation [152]. These tissues may potentially serve as a model to simplify physiological function of an organ. The advanced organs-on-a-chip models are integrated with microsensors, which can detect cells and environmental cues. For instance, to measure the transmembrane electrical resistance across cell barrier and detecting the cell migration [155]. In the near future, this technology can be used for biochemical analysis and biophysical analysis (i.e., tissue mechanics, invasions and fusion). Furthermore, it may also facilitate the testing on molecular diagnostic (i.e., protein, nucleic acid detection).

**Figure 5:**
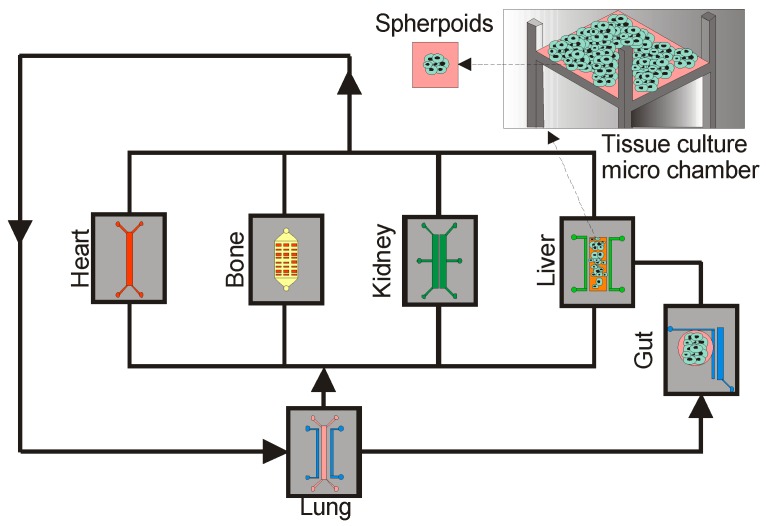
Schematic example of spheroid integration with microfluidic based multiple organ-on-a-chip models.

Many biological processes involve the interaction between multiple organs. Thus, a futuristic organ-on-a-chip model will incorporate multiple organ interactions in physiologically relevant orders. This technology is likely to have a higher accuracy for testing drug metabolism and toxicity for translating basic bench research to clinical practice. This device can be fabricated by using spheroids to further mimic the in-vivo conations of cells and enhance the results [156]. This approach mimics the multiple organ systems in a single device which is termed as a “body-on-a-chip”. Furthermore, the model is designed with a fluid stream, which acts as a network of a surrogate blood vessel to interconnect all tissue compartments biochemically. Thus, the model allows for testing drug effect based on multiple physiological interactions. In the context of drug testing applications, this platform is promising and may increase the screening throughput and facilitate the development of new drug candidates. Further, it allows bioanalysis to identify the pharmacokinetic and pharmacodynamic consequences to predict the safety of the drugs. Since microfluidic cartridges are translucent, they are also useful for time-lapse imaging to identify drug induced pathophysiological changes. Figure 5 shows a schematic presentation the possible use of spheroids to fabricate multi-organ-on-a-chip.

#### Organoids on Chip

Both spheroids based “organ-on-a-chip” and even a “body-on-a-chip” is still in infancy to accurately reproduce and mimic the in vivo niche. Although spheroids consist of organized tissues, the quality of tissues function is limited. Thus, the developments towards the use of organoid cultures have gained attention in the recent years. Organoid can be grown onto a microfluidic platform to model organ features such as development, homeostasis and diseases. These cultures form a higher order tissue organization such as hollow spherical tissue. The organoids are generated using stems cells or embryonic stem. The organoids exclusively represent development system of the embryonic tissue. The stem cells potentially aggregate and assemble into a spatially patterned structure that supports organogenesis. Moreover, organoids also can be developed using induced pluripotent stem (IPS) derived from a patient who represent a personalized cell system that functions as a disease model for an individual.

The organoids are “near physiological” models for drug metabolism and toxicity testing. The development of organoids of the gastrointestinal system also represents an important resource for drug development programs. Initially, intestinal organoids were developed by using stem cells expressing leucine-rich repeat-containing G-protein coupled receptor 5 (Lgr5) [157]. Furthermore, these Lgr5^+^ stem cells can be differentiated and develop organoids of gastrointestinal system (GI) including hepatic system. The organoids of the hepatic system enable the acquisition of biotransformation drugs and toxins to predict drug safety. Recent advances in a microfluidic system can potentially help to miniaturize the hepatic organoids in a single chip for high-throughput screening. For instance, digital microfluidic system was developed for drug testing using arrays of liver organoids [127]. The platform is known as organoid droplet exchange procedure (GODEP), which allows fluid manipulation, e.g. reagent exchange. A continuous media circulation in microfluidic devices is critical for generating growth factor, signaling and drug gradients. However, it is difficult to maintain organoid position in a continuous-flow microfluidic system. Thus a stationary organoid placement could be beneficial for stabilizing tissue position. Recently, liver organoids were encapsulated and loaded in a perfused C-shaped trap arrays. This approach sustains hepatic tissue position during exposure of various fluid flow rates [158].

Organoids are promising candidates for cellular therapeutics. Microfluidics-assisted encapsulation of organoids provides a prominent role in therapeutic delivery systems. For instance, LSFM4LIFE project is an ongoing EU Horizon 2020 program seeks to achieve cellular therapy for type 1 diabetes by using Human Pancreas Organoids (hPOs). Further, alginate-poly-L-lysine is used to encapsulate cells for secreting antibody and therapeutic protein. These microcapsules are called immunotherapeutic organoids. These organoids can be implanted in vivo to deliver therapeutics. They are alternative to drug based treatment for cancer [159] and immunological disorders [160].

**Figure 6:**
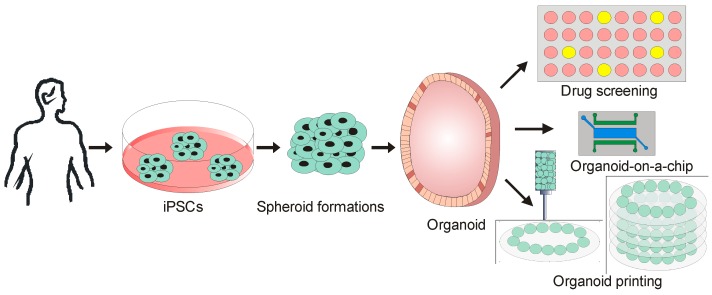
Organoid development and integration with microfluidic technology for drug screening, development organoid-on-a chip model and organoid-based bioprinting.

The integration of biomaterial supports the further development of organoid-based platforms. For instance, patterning of hyaluronic acid (HA) as substrate was used to fabricate 3D contractile cardiac organoids [161]. Recently, researchers at Wake Forest School of Medicine have generated functional cardiac tissue by 3D printing spheroids of cardiac organoids. In another study, hepatic organoids were engineered by assembling thousands of spheroids into a predefined pattern using acoustic nodes [66]. Additionally, Shen et al. demonstrated the construction of human airway epithelial by using epithelial organoids in response to cues from an ECM nano patterned substrate [162]. The applications of IPS-derived organoids are summarized in Figure 6.

## 6. Conclusions

In line with the current trend, 3D cell cultures are cost effective and easy-to-use solutions for various applications. The microfluidic technology evidently demonstrated its suitability for the translation of 3D cell spheroid technology into commercial products. In fact, advances in microfluidics-based spheroid culturing techniques made substantial progress in biomedical ventures including drug screening, tissue engineering, disease modelling and cellular therapeutics. However, further optimization is needed to accurately mimic the in-vivo environment. Ideally, physiologically relevant models will help in overcoming the animal testing and animal based assays. Nonetheless, heed should be given to ensure that these microfluidic tools do not interfere or manipulate the ideal cellular behaviour. Ultimately, the usefulness of iPSC, organoids technology and microfluidic system may serve as a breakthrough for featuring next generation human models. Organoids potentially enhance the similarity of 3D tissue organization to that of real organs. Another interesting direction is the utility of 3D-bioprinted organoids for tissue construct. It is also critical to improve efficacy of drug testing by using this technology. In that regard, an advance look on coupling digitalized organ-on-a-chip systems with mass spectrometry analysis will widen the pipeline of future of drug development. Furthermore, it is crucial to integrate new biosensors technology into multi-organ systems. This is particularly important for feedback and molecular diagnostics. Collectively, the future challenges will be scaling up this technology towards the target for personalized medicine. By this, individualized therapeutic methods can be established as more precise medical approaches for intracellular delivery, cell transplantation and personalized tissue engineering.

## Figures and Tables

**Table 1:** Encapsulation types for 3D cell culture in droplets.

Polymer	Gelation Method	Advantage	Disadvantage
Agarose	Temperature Shift	Improved nutrient diffusion [103] Biocompatible [104]	The gelling temperate must be conducive for optimal cell viability
Gelatin	UV irradiation	Formation cell–matrix interactions with hydrogel [105]	Combination with hydrogel liquefies [106]
Poly(ethylene glycol) (PEG)	UV irradiation	Biodegradable [107]	Poor drug release [107]
lactic-co-glycolic acid (PLGA)	UV irradiation	Release hydrophobic drugs [108]	Poor drug encapsulation [109]
PEG-PLA incorporation	UV irradiation	Ideal for drug delivery [110]	Poor stability [110]
Alginate	Ion reaction	Highly permeable structure and allows long term culture [111]	Rapid gelation process can from non-spherical particles [112]
Pura matrix Hydrogel	Ion reaction	Increase cell attachment, proliferation and differentiation [99]	Decrease the cell viability [113]
Gelatin + Matrigel	Ion reaction	Facilitate cell-assembly [114,115]	The matrigel can induce morphology alteration of the cells [114]

## References

[1] Mahteme H., Lovqvist A., Graf W., Lundqvist H., Carlsson J., Sundin A. (1998). Adjuvant 131i-anti-cea-antibody radioimmunotherapy inhibits the development of experimental colonic carcinoma liver metastases. Anticancer Res..

[2] Sanchez-Romero N., Schophuizen C.M., Gimenez I., Masereeuw R. (2016). In vitro systems to study nephropharmacology: 2D versus 3D models. Eur. J. Pharmacol..

[3] Cukierman E., Pankov R., Stevens D.R., Yamada K.M. (2001). Taking cell-matrix adhesions to the third dimension. Science.

[4] Nath S., Devi G.R. (2016). Three-dimensional culture systems in cancer research: Focus on tumor spheroid model. Pharmacol. Ther..

[5] Ashe H.L., Briscoe J. (2006). The interpretation of morphogen gradients. Development.

[6] Tibbitt M.W., Anseth K.S. (2009). Hydrogels as extracellular matrix mimics for 3D cell culture. Biotechnol. Bioeng..

[7] Liao J., Guo X., Grande-Allen K.J., Kasper F.K., Mikos A.G. (2010). Bioactive polymer/extracellular matrix scaffolds fabricated with a flow perfusion bioreactor for cartilage tissue engineering. Biomaterials.

[8] Moscona A., Moscona H. (1952). The dissociation and aggregation of cells from organ rudiments of the early chick embryo. J. Anat..

[9] Van Duinen V., Trietsch S.J., Joore J., Vulto P., Hankemeier T. (2015). Microfluidic 3D cell culture: From tools to tissue models. Curr. Opin. Biotechnol..

[10] Fu C.Y., Tseng S.Y., Yang S.M., Hsu L., Liu C.H., Chang H.Y. (2014). A microfluidic chip with a U-shaped microstructure array for multicellular spheroid formation, culturing and analysis. Biofabrication.

[11] Nguyen N.T., Shaegh S.A., Kashaninejad N., Phan D.T. (2013). Design, fabrication and characterization of drug delivery systems based on lab-on-a-chip technology. Adv. Drug Deliv. Rev..

[12] Lin L., Chu Y.S., Thiery J.P., Lim C.T., Rodriguez I. (2013). Microfluidic cell trap array for controlled positioning of single cells on adhesive micropatterns. Lab Chip.

[13] Zhu J., Shang J., Olsen T., Liu K., Brenner D., Lin Q. (2014). A mechanically tunable microfluidic cell-trapping device. Sens. Actuators Phys..

[14] Ainla A., Jansson E.T., Stepanyants N., Orwar O., Jesorka A. (2010). A microfluidic pipette for single-cell pharmacology. Anal. Chem..

[15] Wen Y., Yang S.T. (2008). The future of microfluidic assays in drug development. Expert. Opin. Drug Discov..

[16] Ly J., Masterman-Smith M., Ramakrishnan R., Sun J., Kokubun B., van Dam R.M. (2013). Automated reagent-dispensing system for microfluidic cell biology assays. J. Lab Autom..

[17] Gu G.Y., Lee Y.W., Chiang C.C., Yang Y.T. (2015). A nanoliter microfluidic serial dilution bioreactor. Biomicrofluidics.

[18] Alessandri K., Sarangi B.R., Gurchenkov V.V., Sinha B., Kiessling T.R., Fetler L., Rico F., Scheuring S., Lamaze C., Simon A. (2013). Cellular capsules as a tool for multicellular spheroid production and for investigating the mechanics of tumor progression in vitro. Proc. Natl. Acad. Sci. USA.

[19] Sabhachandani P., Motwani V., Cohen N., Sarkar S., Torchilin V., Konry T. (2016). Generation and functional assessment of 3D multicellular spheroids in droplet based microfluidics platform. Lab Chip.

[20] Shield K., Riley C., Quinn M.A., Rice G.E., Ackland M.L., Ahmed N. (2007). α2β1 integrin affects metastatic potential of ovarian carcinoma spheroids by supporting disaggregation and proteolysis. J. Carcinog..

[21] Loessner D., Stok K.S., Lutolf M.P., Hutmacher D.W., Clements J.A., Rizzi S.C. (2010). Bioengineered 3D platform to explore cell-ECM interactions and drug resistance of epithelial ovarian cancer cells. Biomaterials.

[22] Frixen U.H., Behrens J., Sachs M., Eberle G., Voss B., Warda A., Lochner D., Birchmeier W. (1991). E-cadherin-mediated cell-cell adhesion prevents invasiveness of human carcinoma cells. J. Cell Biol..

[23] Marsden M., DeSimone D.W. (2003). Integrin-ecm interactions regulate cadherin-dependent cell adhesion and are required for convergent extension in xenopus. Curr. Biol..

[24] Lin R.Z., Chou L.F., Chien C.C., Chang H.Y. (2006). Dynamic analysis of hepatoma spheroid formation: Roles of e-cadherin and beta1-integrin. Cell Tissue Res..

[25] Takei R., Suzuki D., Hoshiba T., Nagaoka M., Seo S.J., Cho C.S., Akaike T. (2005). Role of e-cadherin molecules in spheroid formation of hepatocytes adhered on galactose-carrying polymer as an artificial asialoglycoprotein model. Biotechnol. Lett..

[26] Haga T., Uchide N., Tugizov S., Palefsky J.M. (2008). Role of E-cadherin in the induction of apoptosis of HPV16-positive CaSki cervical cancer cells during multicellular tumor spheroid formation. Apoptosis.

[27] Ayollo D.V., Zhitnyak I.Y., Vasiliev J.M., Gloushankova N.A. (2009). Rearrangements of the actin cytoskeleton and e-cadherin-based adherens junctions caused by neoplasic transformation change cell-cell interactions. PLoS ONE.

[28] Mueller-Klieser W. (1984). Method for the determination of oxygen consumption rates and diffusion coefficients in multicellular spheroids. Biophys. J..

[29] Mueller-Klieser W. (1984). Microelectrode measurement of oxygen tension distributions in multicellular spheroids cultured in spinner flasks. Recent Results Cancer Res..

[30] Alvarez-Perez J., Ballesteros P., Cerdan S. (2005). Microscopic images of intraspheroidal pH by 1H magnetic resonance chemical shift imaging of pH sensitive indicators. MAGMA.

[31] Hamilton G. (1998). Multicellular spheroids as an in vitro tumor model. Cancer Lett..

[32] Ghosh S., Joshi M.B., Ivanov D., Feder-Mengus C., Spagnoli G.C., Martin I., Erne P., Resink T.J. (2007). Use of multicellular tumor spheroids to dissect endothelial cell–tumor cell interactions: A role for T-cadherin in tumor angiogenesis. FEBS Lett..

[33] Hsiao A.Y., Torisawa Y.S., Tung Y.C., Sud S., Taichman R.S., Pienta K.J., Takayama S. (2009). Microfluidic system for formation of PC-3 prostate cancer co-culture spheroids. Biomaterials.

[34] Patra B., Chen Y.H., Peng C.C., Lin S.C., Lee C.H., Tung Y.C. (2013). A microfluidic device for uniform-sized cell spheroids formation, culture, harvesting and flow cytometry analysis. Biomicrofluidics.

[35] Mehta G., Hsiao A.Y., Ingram M., Luker G.D., Takayama S. (2012). Opportunities and challenges for use of tumor spheroids as models to test drug delivery and efficacy. J. Control Release.

[36] Kato Y., Iwamoto M., Koike T., Suzuki F., Takano Y. (1988). Terminal differentiation and calcification in rabbit chondrocyte cultures grown in centrifuge tubes: Regulation by transforming growth factor beta and serum factors. Proc. Natl. Acad. Sci. USA.

[37] Anada T., Fukuda J., Sai Y., Suzuki O. (2012). An oxygen-permeable spheroid culture system for the prevention of central hypoxia and necrosis of spheroids. Biomaterials.

[38] Jahn K., Richards R.G., Archer C.W., Stoddart M.J. (2010). Pellet culture model for human primary osteoblasts. Eur. Cell Mater..

[39] Giovannini S., Diaz-Romero J., Aigner T., Heini P., Mainil-Varlet P., Nesic D. (2010). Micromass co-culture of human articular chondrocytes and human bone marrow mesenchymal stem cells to investigate stable neocartilage tissue formation in vitro. Eur. Cell Mater..

[40] Ruedel A., Hofmeister S., Bosserhoff A.K. (2013). Development of a model system to analyze chondrogenic differentiation of mesenchymal stem cells. Int. J. Clin. Exp. Pathol..

[41] Ivascu A., Kubbies M. (2006). Rapid generation of single-tumor spheroids for high-throughput cell function and toxicity analysis. J. Biomol. Screen.

[42] Landry J., Bernier D., Ouellet C., Goyette R., Marceau N. (1985). Spheroidal aggregate culture of rat liver cells: Histotypic reorganization, biomatrix deposition, and maintenance of functional activities. J. Cell Biol..

[43] Friedrich J., Ebner R., Kunz-Schughart L.A. (2007). Experimental anti-tumor therapy in 3-D: Spheroids—Old hat or new challenge?. Int. J. Radiat. Biol..

[44] Friedrich J., Seidel C., Ebner R., Kunz-Schughart L.A. (2009). Spheroid-based drug screen: Considerations and practical approach. Nat. Protoc..

[45] Froehlich K., Haeger J.D., Heger J., Pastuschek J., Photini S.M., Yan Y., Lupp A., Pfarrer C., Mrowka R., Schleussner E. (2016). Generation of multicellular breast cancer tumor spheroids: Comparison of different protocols. J. Mammary Gland. Biol. Neoplasia.

[46] Keller G.M. (1995). In vitro differentiation of embryonic stem cells. Curr. Opin. Cell Biol..

[47] Tung Y.C., Hsiao A.Y., Allen S.G., Torisawa Y.S., Ho M., Takayama S. (2011). High-throughput 3D spheroid culture and drug testing using a 384 hanging drop array. Analyst.

[48] Sandu I., Fleaca C.T. (2011). The influence of gravity on the distribution of the deposit formed onto a substrate by sessile, hanging, and sandwiched hanging drop evaporation. J. Colloid. Interface Sci..

[49] Lin B., Miao Y., Wang J., Fan Z., Du L., Su Y., Liu B., Hu Z., Xing M. (2016). Surface tension guided hanging-drop: Producing controllable 3D spheroid of high-passaged human dermal papilla cells and forming inductive microtissues for hair-follicle regeneration. ACS Appl. Mater. Interfaces.

[50] Frey O., Misun P.M., Fluri D.A., Hengstler J.G., Hierlemann A. (2014). Reconfigurable microfluidic hanging drop network for multi-tissue interaction and analysis. Nat. Commun..

[51] Hsiao A.Y., Tung Y.C., Kuo C.H., Mosadegh B., Bedenis R., Pienta K.J., Takayama S. (2012). Micro-ring structures stabilize microdroplets to enable long term spheroid culture in 384 hanging drop array plates. Biomed. Microdevices.

[52] Cavnar S.P., Salomonsson E., Luker K.E., Luker G.D., Takayama S. (2014). Transfer, imaging, and analysis plate for facile handling of 384 hanging drop 3D tissue spheroids. J. Lab Autom..

[53] Nyberg S.L., Hardin J., Amiot B., Argikar U.A., Remmel R.P., Rinaldo P. (2005). Rapid, large-scale formation of porcine hepatocyte spheroids in a novel spheroid reservoir bioartificial liver. Liver Transpl..

[54] Lazar A., Mann H.J., Remmel R.P., Shatford R.A., Cerra F.B., Hu W.S. (1995). Extended liver-specific functions of porcine hepatocyte spheroids entrapped in collagen gel. Cell. Dev. Biol. Anim..

[55] Ingram M., Techy G.B., Saroufeem R., Yazan O., Narayan K.S., Goodwin T.J., Spaulding G.F. (1997). Three-dimensional growth patterns of various human tumor cell lines in simulated microgravity of a nasa bioreactor. Cell. Dev. Biol. Anim..

[56] Khaoustov V.I., Darlington G.J., Soriano H.E., Krishnan B., Risin D., Pellis N.R., Yoffe B. (1999). Induction of three-dimensional assembly of human liver cells by simulated microgravity. Cell. Dev. Biol. Anim..

[57] Sebastian A., Buckle A.M., Markx G.H. (2007). Tissue engineering with electric fields: Immobilization of mammalian cells in multilayer aggregates using dielectrophoresis. Biotechnol. Bioeng..

[58] Okochi M., Matsumura T., Honda H. (2013). Magnetic force-based cell patterning for evaluation of the effect of stromal fibroblasts on invasive capacity in 3D cultures. Biosens. Bioelectron..

[59] Wang T., Green R., Nair R.R., Howell M., Mohapatra S., Guldiken R., Mohapatra S.S. (2015). Surface acoustic waves (SAW)-based biosensing for quantification of cell growth in 2D and 3D cultures. Sensors.

[60] Jaganathan H., Gage J., Leonard F., Srinivasan S., Souza G.R., Dave B., Godin B. (2014). Three-dimensional in vitro co-culture model of breast tumor using magnetic levitation. Sci. Rep..

[61] Haisler W.L., Timm D.M., Gage J.A., Tseng H., Killian T.C., Souza G.R. (2013). Three-dimensional cell culturing by magnetic levitation. Nat. Protoc..

[62] Tseng H., Gage J.A., Raphael R.M., Moore R.H., Killian T.C., Grande-Allen K.J., Souza G.R. (2013). Assembly of a three-dimensional multitype bronchiole coculture model using magnetic levitation. Tissue. Eng. Part C Methods.

[63] Souza G.R., Molina J.R., Raphael R.M., Ozawa M.G., Stark D.J., Levin C.S., Bronk L.F., Ananta J.S., Mandelin J., Georgescu M.M. (2010). Three-dimensional tissue culture based on magnetic cell levitation. Nat. Nanotechnol..

[64] Bazou D., Kuznetsova L.A., Coakley W.T. (2005). Physical enviroment of 2-D animal cell aggregates formed in a short pathlength ultrasound standing wave trap. Ultrasound Med. Biol..

[65] Liu J., Kuznetsova L.A., Edwards G.O., Xu J., Ma M., Purcell W.M., Jackson S.K., Coakley W.T. (2007). Functional three-dimensional HepG2 aggregate cultures generated from an ultrasound trap: Comparison with HepG2 spheroids. J. Cell Biochem..

[66] Chen P., Guven S., Usta O.B., Yarmush M.L., Demirci U. (2015). Biotunable acoustic node assembly of organoids. Adv. Healthc. Mater..

[67] Guo F., Mao Z., Chen Y., Xie Z., Lata J.P., Li P., Ren L., Liu J., Yang J., Dao M. (2016). Three-dimensional manipulation of single cells using surface acoustic waves. Proc. Natl. Acad. Sci. USA.

[68] Chen K., Wu M., Guo F., Li P., Chan C.Y., Mao Z., Li S., Ren L., Zhang R., Huang T.J. (2016). Rapid formation of size-controllable multicellular spheroids via 3D acoustic tweezers. Lab Chip.

[69] Schultz K.M., Furst E.M. (2011). High-throughput rheology in a microfluidic device. Lab Chip.

[70] Yu L., Chen M.C., Cheung K.C. (2010). Droplet-based microfluidic system for multicellular tumor spheroid formation and anticancer drug testing. Lab Chip.

[71] Chen W., Kim J.H., Zhang D., Lee K.H., Cangelosi G.A., Soelberg S.D., Furlong C.E., Chung J.H., Shen A.Q. (2013). Microfluidic one-step synthesis of alginate microspheres immobilized with antibodies. J. R. Soc. Interface.

[72] Tehranirokh M., Kouzani A.Z., Francis P.S., Kanwar J.R. (2013). Microfluidic devices for cell cultivation and proliferation. Biomicrofluidics.

[73] Hu G., Li D. (2007). Three-dimensional modeling of transport of nutrients for multicellular tumor spheroid culture in a microchannel. Biomed. Microdevices.

[74] Wu L.Y., Di Carlo D., Lee L.P. (2008). Microfluidic self-assembly of tumor spheroids for anticancer drug discovery. Biomed. Microdevices.

[75] Wang W.H., Zhang Z.L., Xie Y.N., Wang L., Yi S., Liu K., Liu J., Pang D.W., Zhao X.Z. (2007). Flow-focusing generation of monodisperse water droplets wrapped by ionic liquid on microfluidic chips: From plug to sphere. Langmuir.

[76] Christopher G., Bergstein J., End N., Poon M., Nguyen C., Anna S.L. (2009). Coalescence and splitting of confined droplets at microfluidic junctions. Lab Chip.

[77] Kim L., Toh Y.C., Voldman J., Yu H. (2007). A practical guide to microfluidic perfusion culture of adherent mammalian cells. Lab Chip.

[78] Agastin S., Giang U.B., Geng Y., Delouise L.A., King M.R. (2011). Continuously perfused microbubble array for 3D tumor spheroid model. Biomicrofluidics.

[79] Toh Y.C., Zhang C., Zhang J., Khong Y.M., Chang S., Samper V.D., van Noort D., Hutmacher D.W., Yu H. (2007). A novel 3D mammalian cell perfusion-culture system in microfluidic channels. Lab Chip.

[80] Okuyama T., Yamazoe H., Mochizuki N., Khademhosseini A., Suzuki H., Fukuda J. (2010). Preparation of arrays of cell spheroids and spheroid-monolayer cocultures within a microfluidic device. J.Biosci. Bioeng..

[81] Chen S.Y.C., Hung P.J., Lee P.J. (2011). Microfluidic array for three-dimensional perfusion culture of human mammary epithelial cells. Biomed. Microdevices.

[82] Sakai Y., Hattori K., Yanagawa F., Sugiura S., Kanamori T., Nakazawa K. (2014). Detachably assembled microfluidic device for perfusion culture and post-culture analysis of a spheroid array. Biotechnol. J..

[83] McMillan K.S., Boyd M., Zagnoni M. (2016). Transitioning from multi-phase to single-phase microfluidics for long-term culture and treatment of multicellular spheroids. Lab Chip.

[84] Ruppen J., Cortes-Dericks L., Marconi E., Karoubi G., Schmid R.A., Peng R., Marti T.M., Guenat O.T. (2014). A microfluidic platform for chemoresistive testing of multicellular pleural cancer spheroids. Lab Chip.

[85] Wong K.H., Chan J.M., Kamm R.D., Tien J. (2012). Microfluidic models of vascular functions. Annu. Rev. Biomed. Eng..

[86] Zhu Y., Fang Q. (2013). Analytical detection techniques for droplet microfluidics—A review. Anal. Chim. Acta.

[87] Basova E.Y., Foret F. (2015). Droplet microfluidics in (bio)chemical analysis. Analyst.

[88] Oliveira A.F., Pessoa A.C., Bastos R.G., de la Torre L.G. (2016). Microfluidic tools toward industrial biotechnology. Biotechnol. Prog..

[89] Wen N., Zhao Z., Fan B., Chen D., Men D., Wang J., Chen J. (2016). Development of droplet microfluidics enabling high-throughput single-cell analysis. Molecules.

[90] Martino C., deMello A.J. (2016). Droplet-based microfluidics for artificial cell generation: A brief review. Interface Focus.

[91] Thorsen T., Maerkl S.J., Quake S.R. (2002). Microfluidic large-scale integration. Science.

[92] Clausell-Tormos J., Lieber D., Baret J.C., El-Harrak A., Miller O.J., Frenz L., Blouwolff J., Humphry K.J., Köster S., Duan H. (2008). Droplet-based microfluidic platforms for the encapsulation and screening of mammalian cells and multicellular organisms. Chem. Biol..

[93] Utada A., Lorenceau E., Link D., Kaplan P., Stone H., Weitz D. (2005). Monodisperse double emulsions generated from a microcapillary device. Science.

[94] Utada A., Chu L.-Y., Fernandez-Nieves A., Link D., Holtze C., Weitz D. (2007). Dripping, jetting, drops, and wetting: The magic of microfluidics. MRS Bull..

[95] Cristini V., Renardy Y. (2006). Scalings for droplet sizes in shear-driven breakup: Non-microfluidic ways to monodisperse emulsions. Fluid Dyn. Mater. Process.

[96] Renardy Y. (2007). The effects of confinement and inertia on the production of droplets. Rheol. Acta.

[97] Baret J.C. (2012). Surfactants in droplet-based microfluidics. Lab Chip.

[98] Tumarkin E., Kumacheva E. (2009). Microfluidic generation of microgels from synthetic and natural polymers. Chem. Soc. Rev..

[99] Um E., Lee D.S., Pyo H.B., Park J.K. (2008). Continuous generation of hydrogel beads and encapsulation of biological materials using a microfluidic droplet-merging channel. Microfluid. Nanofluidics.

[100] Tendulkar S., Mirmalek-Sani S.H., Childers C., Saul J., Opara E.C., Ramasubramanian M.K. (2012). A three-dimensional microfluidic approach to scaling up microencapsulation of cells. Biomed. Microdevices.

[101] Chan H.F., Zhang Y., Leong K.W. (2016). Efficient one-step production of microencapsulated hepatocyte spheroids with enhanced functions. Small.

[102] Orive G., Hernandez R.M., Gascon A.R., Calafiore R., Chang T.M., de Vos P., Hortelano G., Hunkeler D., Lacik I., Shapiro A.M. (2003). Cell encapsulation: Promise and progress. Nat. Med..

[103] Eun Y.J., Utada A.S., Copeland M.F., Takeuchi S., Weibel D.B. (2010). Encapsulating bacteria in agarose microparticles using microfluidics for high-throughput cell analysis and isolation. ACS Chem. Biol..

[104] Zamora-Mora V., Velasco D., Hernández R., Mijangos C., Kumacheva E. (2014). Chitosan/agarose hydrogels: Cooperative properties and microfluidic preparation. Carbohydr. Polym..

[105] Mahadik B.P., Haba S.P., Skertich L.J., Harley B.A. (2015). The use of covalently immobilized stem cell factor to selectively affect hematopoietic stem cell activity within a gelatin hydrogel. Biomaterials.

[106] Velasco D., Tumarkin E., Kumacheva E. (2012). Microfluidic encapsulation of cells in polymer microgels. Small.

[107] Nicodemus G.D., Bryant S.J. (2008). Cell encapsulation in biodegradable hydrogels for tissue engineering applications. Tissue Eng. Part B Rev..

[108] Hsu M.N., Luo R., Kwek K.Z., Por Y.C., Zhang Y., Chen C.H. (2015). Sustained release of hydrophobic drugs by the microfluidic assembly of multistage microgel/poly (lactic-co-glycolic acid) nanoparticle composites. Biomicrofluidics.

[109] Karnik R., Gu F., Basto P., Cannizzaro C., Dean L., Kyei-Manu W., Langer R., Farokhzad O.C. (2008). Microfluidic platform for controlled synthesis of polymeric nanoparticles. Nano. Lett..

[110] Shum H.C., Kim J.W., Weitz D.A. (2008). Microfluidic fabrication of monodisperse biocompatible and biodegradable polymersomes with controlled permeability. J. Am. Chem. Soc..

[111] Choi C.H., Jung J.H., Rhee Y.W., Kim D.P., Shim S.E., Lee C.S. (2007). Generation of monodisperse alginate microbeads and in situ encapsulation of cell in microfluidic device. Biomed. Microdevices.

[112] Akbari S., Pirbodaghi T. (2014). Microfluidic encapsulation of cells in alginate particles via an improved internal gelation approach. Microfluid. Nanofluidics.

[113] Kim M.S., Yeon J.H., Park J.K. (2007). A microfluidic platform for 3-dimensional cell culture and cell-based assays. Biomed. Microdevices.

[114] Tamura M., Yanagawa F., Sugiura S., Takagi T., Sumaru K., Kanamori T. (2015). Click-crosslinkable and photodegradable gelatin hydrogels for cytocompatible optical cell manipulation in natural environment. Sci. Rep..

[115] Wang Y., Wang J. (2014). Mixed hydrogel bead-based tumor spheroid formation and anticancer drug testing. Analyst.

[116] Shi X., Sun L., Jiang J., Zhang X., Ding W., Gan Z. (2009). Biodegradable polymeric microcarriers with controllable porous structure for tissue engineering. Macromol. Biosci..

[117] Wang J., Cheng Y., Yu Y., Fu F., Chen Z., Zhao Y., Gu Z. (2015). Microfluidic generation of porous microcarriers for three-dimensional cell culture. ACS Appl. Mater. Interfaces.

[118] Li T., Zhao L., Liu W., Xu J., Wang J. (2016). Simple and reusable off-the-shelf microfluidic devices for the versatile generation of droplets. Lab Chip.

[119] Suzuki K., Homma H., Murayama T., Fukuda S., Takanobu H., Miura H. (2010). Electrowetting-based actuation of liquid droplets for micro transportation systems. J.Adv. Mech. Des. Syst. Manuf..

[120] Pollack M.G., Fair R.B., Shenderov A.D. (2000). Electrowetting-based actuation of liquid droplets for microfluidic applications. Appl. Phys. Lett..

[121] Barbulovicnad I., Yang H., Park P.S., Wheeler A.R. (2008). Digital microfluidics for cell-based assays. Lab Chip.

[122] Cho S.K., Moon H., Kim C.J. (2003). Creating, transporting, cutting, and merging liquid droplets by electrowetting-based actuation for digital microfluidic circuits. J. Microelectromech. Syst..

[123] Griffth E.J., Akella S., Goldberg M.K. (2006). Performance characterization of a reconfigurable planar array digital microfluidic system. Design Automation Methods and Tools for Microfluidics-Based Biochips.

[124] Yoon J.Y., Garrell R.L. (2003). Preventing biomolecular adsorption in electrowetting-based biofluidic chips. Anal. Chem..

[125] Hong J., Kim Y.K., Won D.J., Kim J., Lee S.J. (2015). Three-dimensional digital microfluidic manipulation of droplets in oil medium. Sci. Rep..

[126] Fiddes L.K., Luk V.N., Au S.H., Ng A.H., Luk V., Kumacheva E., Wheeler A.R. (2012). Hydrogel discs for digital microfluidics. Biomicrofluidics.

[127] Au S.H., Chamberlain M.D., Mahesh S., Sefton M.V., Wheeler A.R. (2014). Hepatic organoids for microfluidic drug screening. Lab Chip.

[128] Aijian A.P., Garrell R.L. (2015). Digital microfluidics for automated hanging drop cell spheroid culture. J. Lab Autom..

[129] Vadivelu R.K., Ooi C.H., Yao R.Q., Velasquez J.T., Pastrana E., Diaz-Nido J., Lim F., Ekberg J.A., Nguyen N.T., St John J.A. (2015). Generation of three-dimensional multiple spheroid model of olfactory ensheathing cells using floating liquid marbles. Sci. Rep..

[130] Sarvi F., Arbatan T., Chan P.P.Y., Shen W. (2013). A novel technique for the formation of embryoid bodies inside liquid marbles. Rsc. Adv..

[131] Oliveira N.M., Correia C.R., Reis R.L., Mano J.F. (2015). Liquid marbles for high-throughput biological screening of anchorage-dependent cells. Adv. Healthc. Mater..

[132] Ooi C.H., Van Nguyen A., Evans G.M., Gendelman O., Bormashenko E., Nguyen N.T. (2015). A floating self-propelling liquid marble containing aqueous ethanol solutions. RSC Adv..

[133] Khaw M.K., Ooi C.H., Mohd-Yasin F., Vadivelu R., John J.S., Nguyen N.T. (2016). Digital microfluidics with a magnetically actuated floating liquid marble. Lab Chip.

[134] Han X., Lee H.K., Lim W.C., Lee Y.H., Phan-Quang G.C., Phang I.Y., Ling X.Y. (2016). Spinning liquid marble and its dual applications as microcentrifuge and miniature localized viscometer. ACS Appl. Mater. Interfaces.

[135] Castro J.O., Neves B.M., Rezk A.R., Eshtiaghi N., Yeo L.Y. (2016). Continuous production of Janus and composite liquid marbles with tunable coverage. ACS Appl. Mater. Interfaces.

[136] Enger J., Goksör M., Ramser K., Hagberg P., Hanstorp D. (2004). Optical tweezers applied to a microfluidic system. Lab Chip.

[137] Grier D.G. (2003). A revolution in optical manipulation. Nature.

[138] Chiou P.Y., Chang Z., Wu M.C. (2008). Droplet manipulation with light on optoelectrowetting device. J. Microelectromech. Syst..

[139] Park S.Y., Chiou P.Y. (2011). Light-driven droplet manipulation technologies for lab-on-a-chip applications. Adv. OptoElectron..

[140] Baigl D. (2012). Photo-actuation of liquids for light-driven microfluidics: State of the art and perspectives. Lab Chip.

[141] Jiang D., Park S.Y. (2016). Light-driven 3D droplet manipulation on flexible optoelectrowetting devices fabricated by a simple spin-coating method. Lab Chip.

[142] Pandiyan V.P., John R. (2016). Optofluidic bioimaging platform for quantitative phase imaging of lab on a chip devices using digital holographic microscopy. Appl. Opt..

[143] Shin D., Daneshpanah M., Anand A., Javidi B. (2010). Optofluidic system for three-dimensional sensing and identification of micro-organisms with digital holographic microscopy. Opt. Lett..

[144] Jagannadh V.K., Adhikari J.V., Gorthi S.S. (2015). Automated cell viability assessment using a microfluidics based portable imaging flow analyzer. Biomicrofluidics.

[145] Moldovan N.I., Hibino N., Nakayama K. (2016). Principles of the *Kenzan* method for robotic cell spheroid-based 3D bioprinting. Tissue Eng. Part B Rev..

[146] Mehesz A.N., Brown J., Hajdu Z., Beaver W., da Silva J.V., Visconti R.P., Markwald R.R., Mironov V. (2011). Scalable robotic biofabrication of tissue spheroids. Biofabrication.

[147] Kudan Y.V., Pereira F.D., Parfenov V.A., Kasyanov V.A., Khesuani Y.D., Bulanova Y.A., Mironov V.A. (2015). spreading of tissue spheroids from primary human fibroblasts on the surface of microfibrous electrospun polyurethane matrix (a scanning electron microscopic study). Morfologiia.

[148] Whatley B.R., Li X., Zhang N., Wen X. (2014). Magnetic-directed patterning of cell spheroids. J. Biomed. Mater. Res. A.

[149] Leonard F., Godin B. (2016). 3D in vitro model for breast cancer research using magnetic levitation and bioprinting method. Methods Mol. Biol..

[150] Susienka M.J., Wilks B.T., Morgan J.R. (2016). Quantifying the kinetics and morphological changes of the fusion of spheroid building blocks. Biofabrication.

[151] Munaz A., Vadivelu R.K., John J.A., Nguyen N.T. (2016). A lab-on-a-chip device for investigating the fusion process of olfactory ensheathing cell spheroids. Lab Chip.

[152] Maschmeyer I., Lorenz A.K., Schimek K., Hasenberg T., Ramme A.P., Hubner J., Lindner M., Drewell C., Bauer S., Thomas A. (2015). A four-organ-chip for interconnected long-term co-culture of human intestine, liver, skin and kidney equivalents. Lab.Chip.

[153] Esch M.B., Smith A.S., Prot J.M., Oleaga C., Hickman J.J., Shuler M.L. (2014). How multi-organ microdevices can help foster drug development. Adv. Drug Deliv. Rev..

[154] Materne E.M., Ramme A.P., Terrasso A.P., Serra M., Alves P.M., Brito C., Sakharov D.A., Tonevitsky A.G., Lauster R., Marx U. (2015). A multi-organ chip co-culture of neurospheres and liver equivalents for long-term substance testing. J. Biotechnol..

[155] Bhatia S.N., Ingber D.E. (2014). Microfluidic organs-on-chips. Nat. Biotechnol..

[156] Materne E.M., Maschmeyer I., Lorenz A.K., Horland R., Schimek K.M., Busek M., Sonntag F., Lauster R., Marx U. (2015). The multi-organ chip—A microfluidic platform for long-term multi-tissue coculture. J. Vis. Exp..

[157] Kretzschmar K., Clevers H. (2016). Organoids: Modeling development and the stem cell niche in a dish. Dev. Cell.

[158] Schepers A., Li C., Chhabra A., Seney B.T., Bhatia S. (2016). Engineering a perfusable 3D human liver platform from iPS cells. Lab Chip.

[159] Saenz D.B.L., Compte M., Aceves M., Hernandez R.M., Sanz L., Alvarez-Vallina L., Pedraz J.L. (2015). Microencapsulation of therapeutic bispecific antibodies producing cells: Immunotherapeutic organoids for cancer management. J. Drug Target.

[160] Yang Y., Opara E.C., Liu Y., Atala A., Zhao W. (2017). Microencapsulation of porcine thyroid cell organoids within a polymer microcapsule construct. Exp. Biol. Med..

[161] Khademhosseini A., Eng G., Yeh J., Kucharczyk P.A., Langer R., Vunjak-Novakovic G., Radisic M. (2007). Microfluidic patterning for fabrication of contractile cardiac organoids. Biomed. Microdevices.

[162] Shen Y., Hou Y., Yao S., Huang P., Yobas L. (2015). In vitro epithelial organoid generation induced by substrate nanotopography. Sci. Rep..

